# A near-wearless and extremely long lifetime amorphous carbon film under high vacuum

**DOI:** 10.1038/srep11119

**Published:** 2015-06-10

**Authors:** Liping Wang, Renhui Zhang, Ulf Jansson, Nils Nedfors

**Affiliations:** 1 State Key Laboratory of Solid Lubrication, Lanzhou Institute of Chemical Physics, Chinese Academy of Science, Lanzhou 730000, China; 2 University of Chinese Academy of Sciences, Beijing 100039, China; 3 Department of Materials Chemistry, The Ångström Laboratory, Uppsala University, box 538, SE-751 21 Uppsala, Sweden

## Abstract

Prolonging wear life of amorphous carbon films under vacuum was an enormous challenge. In this work, we firstly reported that amorphous carbon film as a lubricant layer containing hydrogen, oxygen, fluorine and silicon (a-C:H:O:F:Si) exhibited low friction (~0.1), ultra-low wear rate (9.0 × 10^–13^ mm^3^ N^–1^ mm^–1^) and ultra-long wear life (>2 × 10^6^ cycles) under high vacuum. We systematically examined microstructure and composition of transfer film for understanding of the underlying frictional mechanism, which suggested that the extraordinarily excellent tribological properties were attributed to the thermodynamically and structurally stable FeF_2_ nanocrystallites corroborated using first-principles calculations, which were induced by the tribochemical reaction.

Amorphous carbon films have attracted enormous attention in recent years due to their superiorly physical and chemical properties^1^. In particular, super-low friction originated from weak shearing force between hydrogen atoms under vacuum was of remarkable interest^2^. However, wear life of these carbon films was frequently overlooked. Obtaining super-low friction inevitably induced decreasing wear life, wear life of these films ranged from several hundreds to 10^5^ cycles in vacuum as declared by previous study^3^,^4^,^5^,^6^, which was attributed to fast failure of amorphous carbon films under vacuum. The initial H-passivated surfaces of amorphous carbon film were quickly replaced by carbon-rich transfer layers under vacuum due to easily damage of C–H bonds. Then the ultimate friction occurred between two carbonaceous surfaces resulting in high friction and severe wear under high vacuum due to strong cross-interface carbon–carbon interactions in case that dangling bonds of carbon atoms at the interfaces are not sufficiently passivated^7^. Accordingly, although the hydrogenated amorphous carbon film exhibited ultra-long wear life about 1.75 × 10^7^ cycles in dry N_2_^8^ and wear rate below 10^−12^ mm^3^ N^−1^ mm^−1^ (near-wearless^9^), wear life beyond 10^5^ sliding cycles have been believed to be impossible to achieve for amorphous carbon films under vacuum. Based on these previous researches, even though Ag or Al doped amorphous carbon films could improve the tribological performances under vacuum, to some extent^10^,^11^. But the aim of prolonging wear life of these carbon films were still not well achieved. Thus, given that the further and potential space applications of these carbon films, extending wear life and improving wear resistance was imperative.

In this study, we disclosed a Si and F codoped amorphous carbon film. According to our previous studies, Si element played an important role in reducing the internal stress of the carbon film^12^,^13^. And Yang *et al.* declared that for Si-incorporated amorphous carbon films, high and unstable friction was observed under vacuum^14^. Indeed, in this paper, Si doped amorphous carbon film exhibited poor tribological properties under vacuum (see Figure S1 in Supporting Information). Thus, we focused on investigating the influence of fluorine on friction properties of a-C:H:O:F:Si film under vacuum. The results showed that the amorphous carbon film, which exhibited low friction (~0.1), ultra-low wear (9.0 × 10^–13^ mm^3^ N^–1^ mm^–1^) and ultra-long life (>2 × 10^6^ cycles), was firstly reported under high vacuum. The microstructure and composition of transfer film were discussed in details using XPS, EELS and TEM for understanding of the underlying friction mechanism. Results for low friction and wear depended on the formation of FeF_2_ nanocrystallites that induced by tribochemical reaction during sliding. According to the first-principles calculations, the formation of FeF_2_ was thermodynamically and structurally stable, which ensured the superior tribological properties under high vacuum.

## Results

**Figure 1:**
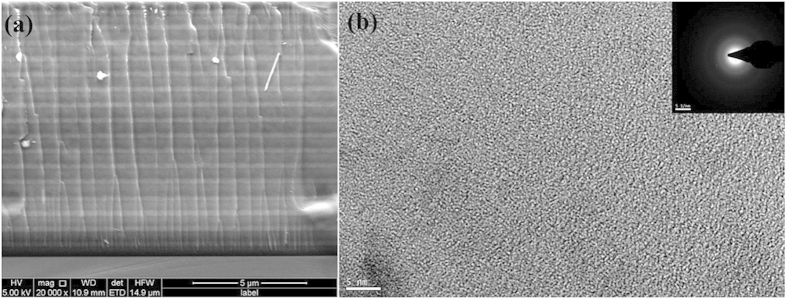
(**a**) SEM cross-sectional image of amorphous carbon coating. (**b**) High resolution TEM (HRTEM) image of amorphous carbon coating (inset is SAED pattern).

The typical carbon film with a total thickness of about 11 ± 0.2 μm, including a silicon adhesive layer (0.2 ± 0.03 μm), a-C:H:O:F:Si layers (0.2 ± 0.02 or 0.5 ± 0.04 μm), and amorphous carbon layer (0.3 ± 0.03 μm), consists of a-C:H:O:F:Si layers placed on top of Si adhesive layer, covered by an amorphous carbon layer, presented in Figure 1a, the detailed deposition processes can be shown in Figure S3. Figure 1b shows HRTEM image of coating surface. It exhibits an amorphous carbon network structure according to the SAED patterns shown in inset image in Figure 1b.

**Figure 2:**
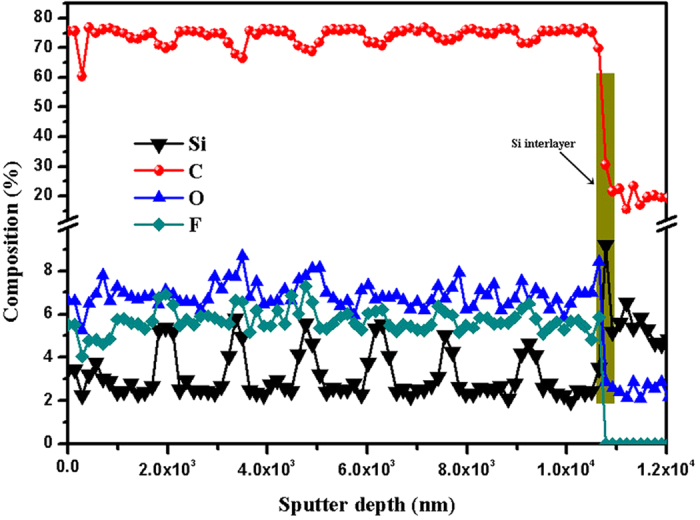
AES spectra of the amorphous carbon film.

The composition of the amorphous carbon film is estimated by Auger electron spectroscopy (AES), as shown in Figure 2C is the major composition of the film. The rest elements are Si, O and F. The hydrogen concentration is examined by time-of-flight elastic recoil detection analysis as shown in Figure S4.

**Figure 3:**
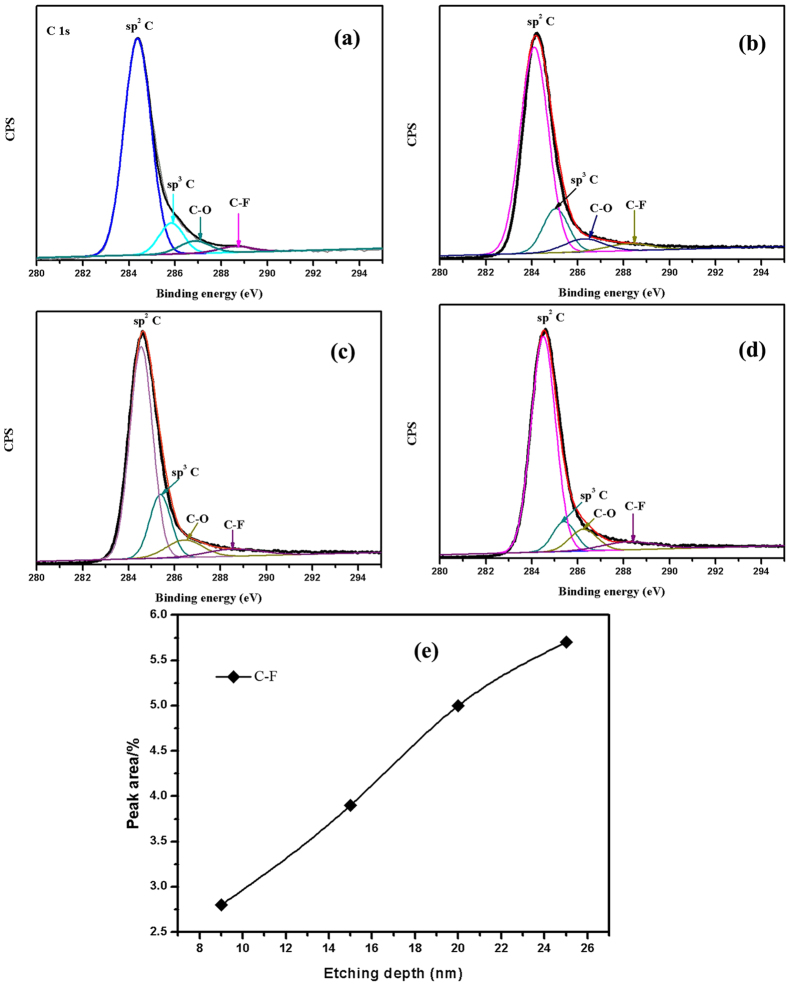
Deconvoluted XPS C 1s spectra of amorphous carbon film as a function of etching depth: (**a**) 9 nm, (**b**) 15 nm, (**c**) 20 nm, (**d**) 25 nm, and (**e**) The fitted peak area as a function of etching depth.

The composition of the uppermost part of the amorphous carbon film is further investigated by sputter profiles carried out by XPS acquired by Ar^+^ sputtering at 3.2 kV. The deconvoluted XPS C 1 s spectra of amorphous carbon film as a function of etching depth are shown in Figure 3a–d. The C 1s peaks can be fit to peaks at 284.2 ± 0.3, 285.3 ± 0.1, 286.5 ± 0.1 eV, and 288.5 ± 0.1 eV and thus assigned to sp^2^ hybrids forms of carbon, sp^3^ hybrids forms of carbon, C–O bonds, and C–F bonds, respectively^15^,^16^. In addition, Figure 3e shows the fitted peak area as a function of etching depth. From Figure 3e, it can be seen that the peak area of C–F bonds tends to increase with increasing etching depth. The result of XPS analysis is consistent with the AES analysis. The hardness and elastic modulus values as a function of depth of indent determined by nanoindentation are presented in Figure S5. The values level off at the final stage of indentation proves to be no substrate contribution to the measured hardness and elastic modulus. The amorphous carbon film has high hardness and elastic modulus values of 14 and 174 GPa, which exhibits the diamond-like feature of this film. In scratch method, as shown in the typical scratch curve in Figure S6, the peeling-off value (about 27 N) merely meant that the film detached from the substrate, that was obviously adhesion failure mode^17^. The critical adhesion load is more than 20 N, indicating the well adhesion between film and substrate, which is attributed to the low internal stress of the film (about −0.55 GPa).

**Figure 4:**
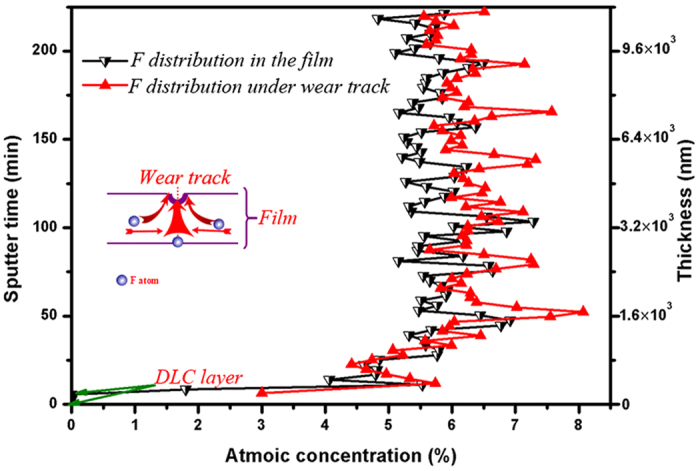
AES spectra of F distribution in the original film and under the wear track as a function of sputter time and thickness, inset is the move trajectory of F atoms under wear track during sliding.

Rangan reported that FeF_2_ nanocrystallites could be obtained by exposing metallic Fe to XeF_2_^18^, which was attributed to the strong electronegativity of F atom^19^. And Jeng and Diebel declared that F showed anomalous diffusion behavior^20^,^21^. As shown in Figure 4, F is redistributed during sliding resulting in the competition among the escape, capture, and recombination processes.

**Figure 5:**
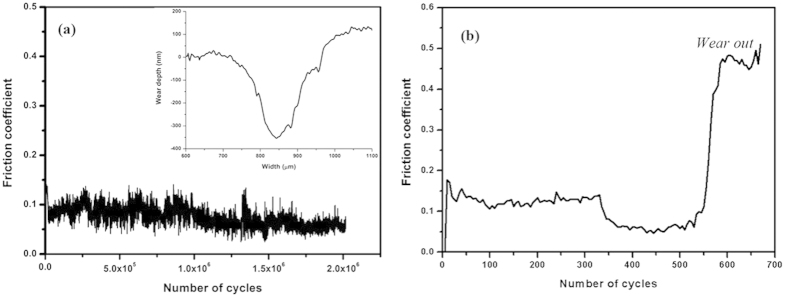
A frictional curve of (**a**) a-C:H:F:Si film (the inset image is the cross-sectional profiles of the wear track) and (b) a-C:H film against steel ball under high vacuum.

Figure 5a shows the friction coefficient of a-C:H:O:F:Si film as a function of sliding cycles (another two repeat experimental results see Figures S7a, S7b and Table S1). The friction coefficient reaches a low state (<0.1) after a very short running-in period (about 40 sliding cycles), then the friction coefficient is yet kept at about 0.1. The inset image of Figure 5a presents the cross-sectional profile of the wear tack detected by a Micro-XAM 3D surface profiler (ADE Phase Shift, USA) after two million sliding cycles. The wear track exhibits depth of only around 350 nm, indicating ultra-low wear (<1.75 × 10^–4^ nm/cycle) during the whole periods. In addition, it is worth noting that the coating exhibits ultra-low wear rate (9.0 × 10^–13^ mm^3^ N^–1^ mm^–1^) and ultra-long lifetime (>2 × 10^6^ cycles) in vacuum. The friction coefficient curve of a-C:H film is presented in Figure 5b. It shows that the film merely maintains low friction within 550 sliding cycles, then is worn out. This fact confirms that the addition of F is helpful for the improvement of vacuum tribological properties.

**Figure 6:**
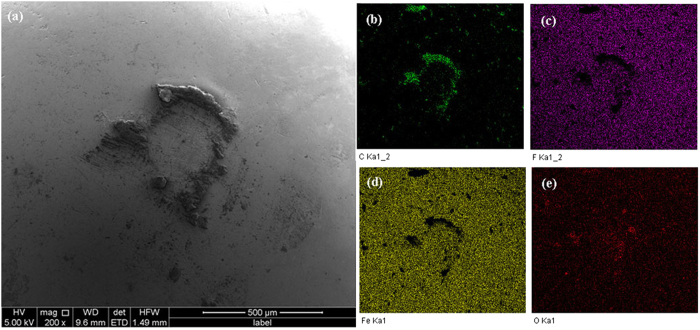
(**a**) Secondary electron image of the steel ball surface after the sliding test against the amorphous carbon film for 2400 sliding cycles. The elemental EDS maps taken from the whole area on (**a**) are shown for (**b**) C, (**c**) F, (**d**) Fe and (**e**) O on the steel ball surface.

A SEM examination of the steel ball surface (Figure 6a) that is contact with the amorphous carbon film, combining with EDS elemental maps taken from the surface (Figure 6b–e) indicate that the steel ball surface is plastically deformed. The carbonaceous material is detected around the wear scar of steel ball. Some oxygen associates with C/ or Fe is also presented (Figure 6e). F is distributed on the whole steel ball surface.

**Figure 7:**
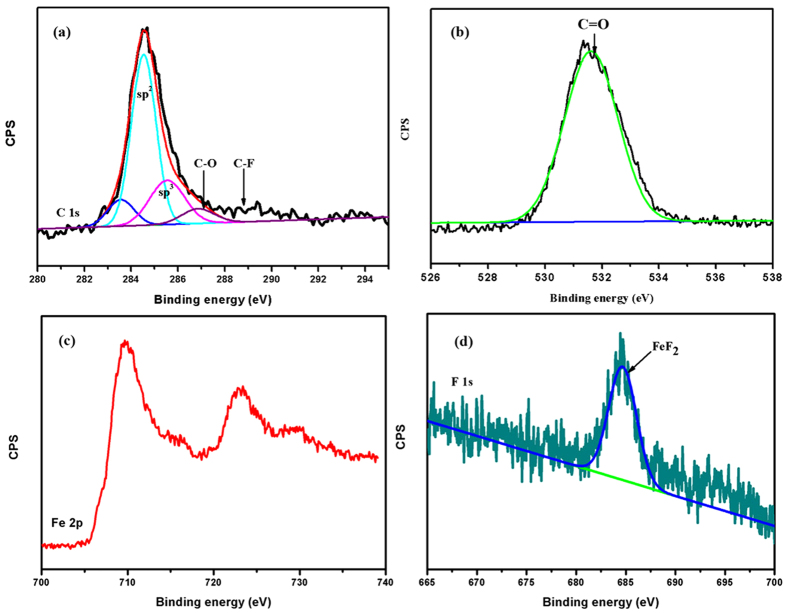
XPS spectra (**a**) C 1s, (**b**) O 1s, (**c**) Fe 2p and (**d**) F1s of transfer film for 2400 sliding cycles.

**Figure 8:**
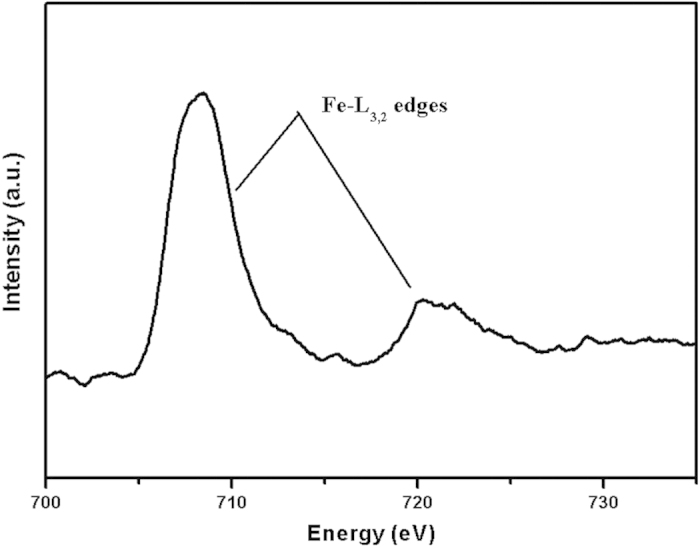
Electron energy loss spectra of the transfer film.

As shown in Figure 7a, the fitting C1s spectra of the transfer film allows discrimination of these five components at the binding energies 283.5 eV, 284.5 eV, 285.5 eV, 286.6 eV and 288.7 eV corresponding to Si–C bonds, sp^2^ carbon bonds, sp^3^ carbon bonds, C–O bonds and C–F bonds, respectively^15^,^16^,^22^,^23^. The transfer film is mainly composed of sp^2^ hybrids forms of carbon. The fitting O 1s spectra presented in Figure 7b shows that it allows discrimination of one peak at the binding energy 531.6 eV corresponding to C = O bonds^24^. The peak of Fe 2p_3/2_ located at 710.6 eV, assigned to FeF_2_^25^, shown in Figure 7c, and the native oxide formed on steel ball surface was absent between steel ball and transfer film interface. In general, the native iron oxide layer was possibly removed during sliding, which may allow the nascent Fe to react with F atoms forming FeF_2_. Indeed, the peak of FeF_2_ (684.9 eV^26^) is found, as shown in Figure 7d. Even so, it is essential to evaluate the presence of any FeF_3_ due to almost the same binding energy (FeF_3_: 685.0 eV^27^). In addition, electron energy loss spectroscopy (EELS) presented in Figure 8 and XPS spectra of F 1s and Fe 2p rule out the presence of FeF_3_.

**Figure 9:**
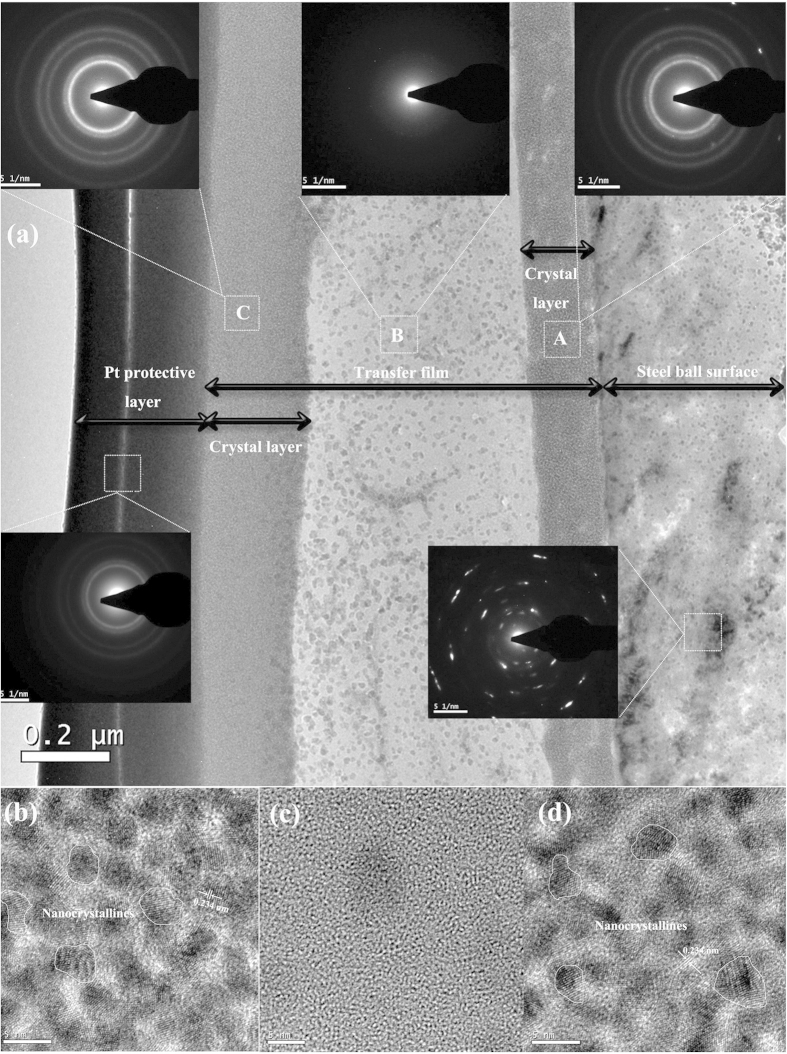
(**a**) TEM image of the interface between the transfer film and the steel ball surface for 2 × 10^6^ sliding cycles. (**b**) The High Resolution TEM (HR TEM) image of the inner F-rich crystal layer in transfer film marked as “A”. (**c**) The HR TEM image of an amorphous carbon layer in transfer film marked as “B”. (**d**) The HRTEM image of the outer F-rich crystal layer in transfer film marked as “C”.

**Figure 10:**
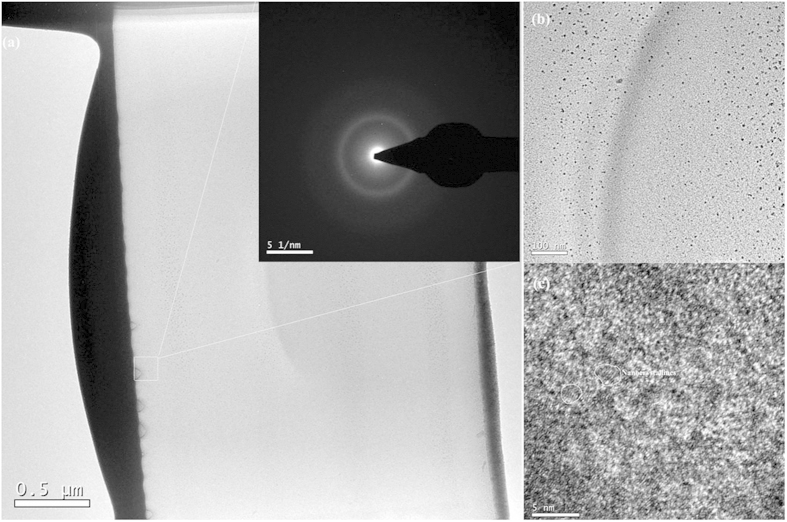
(**a**) TEM images of wear track slice after 2 × 10^6^ sliding cycles. (**b**) High Resolution TEM (HR TEM) image of the marked region in Fig. 12a.

**Figure 11:**
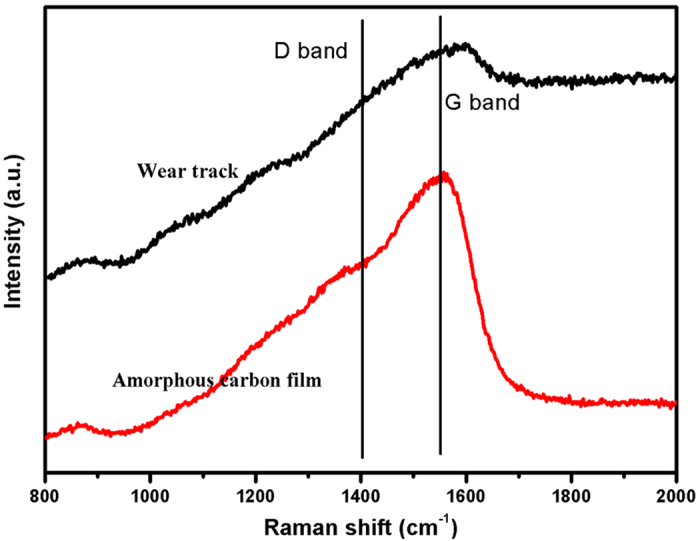
Raman spectra of the amorphous carbon film and wear track.

Thereafter, the transfer film after 2 × 10^6^ sliding cycles is investigated, as shown in Figure 9. Figure 9a shows a TEM image of the transfer film after 2 × 10^6^ sliding cycles. According to the SAED patterns in Figure 9a, the transfer film is consisted of the inner crystal layer (designated as “A”), amorphous carbon layer (designated as “B”) and the outer crystal layer (designated as “C”). The inner crystal layer about 0.15 μm between the steel ball and transfer film interface is presented in Figure 9b, which indicates that when steel ball slides against amorphous carbon film, the F reacts with Fe and forms FeF_2_ nanocrystallites with a d-spacing of 0.234 nm on the steel ball surface. An HR TEM image (Figure 9c) of the transfer film’s mid-section (“B” in Figure 9a) shows that this transfer layer is an amorphous structure attributed to carbon. The topmost part of the transfer film in contact with amorphous carbon film is richer in F compared to the mid-section transfer layer. Figure 9d (“C” in Figure 9a) shows that the thickness of this layer is about 0.2 μm and contains packed ultra-fine nanocrystallites. The selected area diffraction pattern obtained from this region is shown in the inset in Figure 9a, which indicates that FeF_2_ nanocrystallites are also formed during sliding in the out crystal layer with a d-spacing of 0.234 nm. The TEM image of the wear track after 2 × 10^6^ sliding cycles is shown in Figure 10. Figure 10a presents the TEM image of wear track slice after 2 × 10^6^ sliding cycles. The selected area diffraction pattern obtained from the marked region in Figure 10a shows that the selected area is a crystallites-containing amorphous structure, as confirmed by Figure 10b, which has large amount of nanocrystallites in the selected area with d-spacings of 0.202 and 0.234 nm corresponding to Fe (1 1 0) and FeF_2_ (1 1 1) planes. Subsequently, the microstructure of the amorphous carbon film surface and wear track after 2 × 10^6^ sliding cycles is detected by Raman spectra presented in Figure 11. After tribological test, the D peak becomes more pronounced and the position of G peak shifts to higher Raman frequency, indicating significant graphitization of amorphous carbon film^28^,^29^.

**Figure 12:**
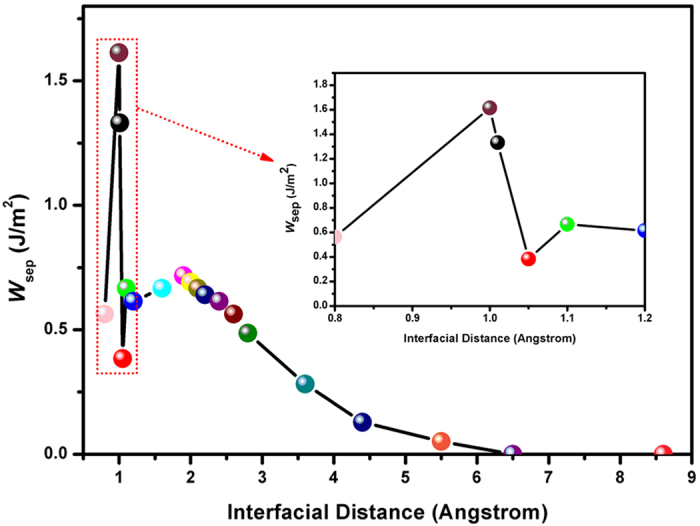
Universal binding energy curves for the Fe and FTD interface, the minimum energy refers to the equilibrium distance between Fe and FTD layers.

The stability of FeF_2_ induced by tribochemical reaction could ensure the superior tribological properties of the film under high vacuum. Subsequently, first-principles calculations are selected to probe the stability of FeF_2_. The total energies of a relaxed interface are calculated with different interfacial separation *d*_Fe–F_ (0.8–8.6 Å). Then the relationship of work of separation (*W*_sep_) versus interfacial separation *d*_Fe–F_ is determined, which is called the universal binding energy relation (UBER, shown in Figure 12). For Fe and FTD interface, the decrease in *W*_sep_ is not continuous, but incremental decreases in *W*_sep_ are followed by local maxima. The *W*_sep_ initially reaches a value of 0.05 J m^−2^ at *d*_Fe–F_ = 5.5 Å, above which *W*_sep_ starts to increase–indicating repulsion between Fe and FTD until to *d*_Fe–F_ = 1.01 Å. At *d*_Fe–F_ = 1.9 Å, *W*_sep_ increases to a local maximum of 0.71 J m^−2^ and then increases to another local maximum value of 1.33 J m^−2^ at *d*_Fe–F_ = 1.01 Å. For *d*_Fe–F_ = 1.01 Å, this interface has the Fe–F bond length of 1.745 Å that indicates the strong bonding between the adjacent Fe and F atoms. As is evident from Figure 12, the strongest cohesion between Fe and F atom is exhibited by the interface distance at 1.0 Å, the F-terminated interface with the interfacial F atoms lies on top of Fe surface. The largest *W*_sep_ for this interface, *W*_sep_ = 1.61 J m^−2^, is explained by the presence of four strong Fe–F bonds with the bond length of 1.743 Å which is close to Fe surface. Oleinik illustrated that positive *W*_sep_ implied that the interfaces were thermodynamically and structurally stable^30^. Thus, this fact is confirmed that interface structures at *d*_Fe–F_ = 1.01 or 1.0 Å are thermodynamically and structurally stable.

**Figure 13:**
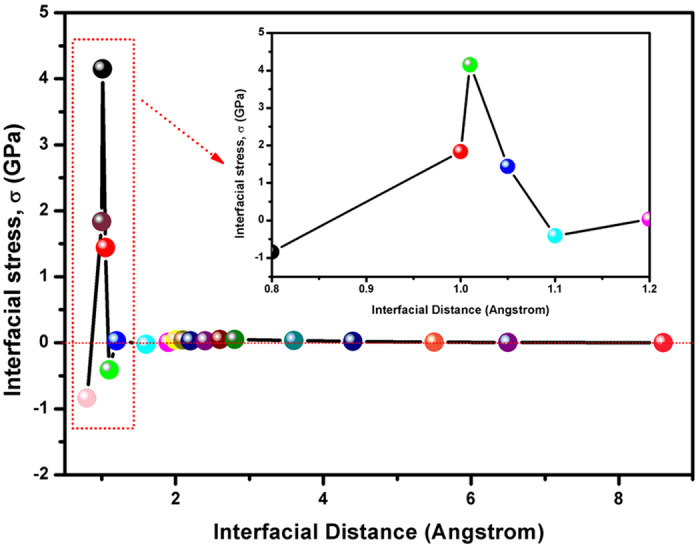
The variations in the interfacial distance, *d*_Fe–F_ at the Fe and FTD with the stress, σ, applied at the interface.

**Figure 14:**
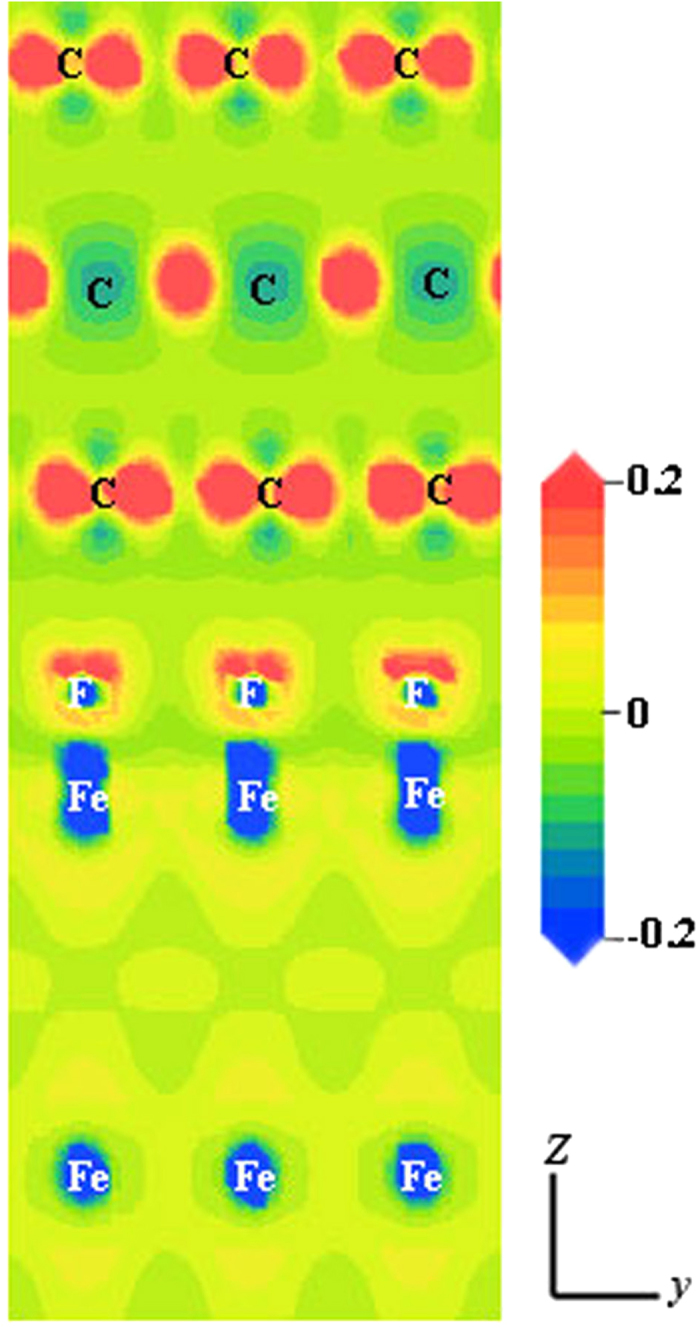
Charge density difference along the relaxed interface calculated with GGA approach.

Assuming that the process of causing the surfaces closer is achieved by applying an external pressure to the interfaces, the stress in the z–direction, σ, normal to interface plane can be calculated using:
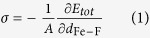
where *A*, the area of interface plane, is equal to 6.25 Å^2^. The stress can be considered as the contact pressure on the Fe and FTD interfaces. As shown in Figure 13, the interfacial stress is around 0 GPa at the *d*_Fe-F_ range from 1.2 to 8.6 Å. It exhibits that the weak reaction between Fe and FTD surface could be neglected. This is not in contradiction with analysis of the results of work of separation. For *d*_Fe−F_ at 0.8 and 1.1 Å, the interfacial stress is about –0.8 and –0.4 GPa, indicating compression between the Fe and FTD surface^31^. The one F atom transfers from the FTD surface to the Fe surface when σ sets as 4.1 or 1.8 GPa. The calculated results reveal that the adhesion transfer of F atom could occur when the distance *d*_Fe–F_ at 1.0 or 1.1 Å. After relaxation, the charge density difference of the interface is given in Figure 14. In the first layer of Fe side, a wide range of charge depletion region exists. The charge distribution, which transfers to the interface region, helps to the formation of chemical bonds between the interfaces. For Fe and FTD interface, the atoms of Fe side lose more charges, which transfer to the interface. As the electronegative ability of F atom is strong, the electrons at the interface shift to the F atoms side. This observation indicates that the charge depletion and accumulation region simultaneously exists at the interface leading to forming a sharp polar ionic bond between Fe and F atoms. Figure S8 exhibits the formation of FeF_2_ is thermodynamically and structurally stable. It guaranteed the superior tribological properties under vacuum due to the excellent anti-wear performance of FeF_2_ as declared in our previous study^32^.

## Discussion

**Figure 15:**
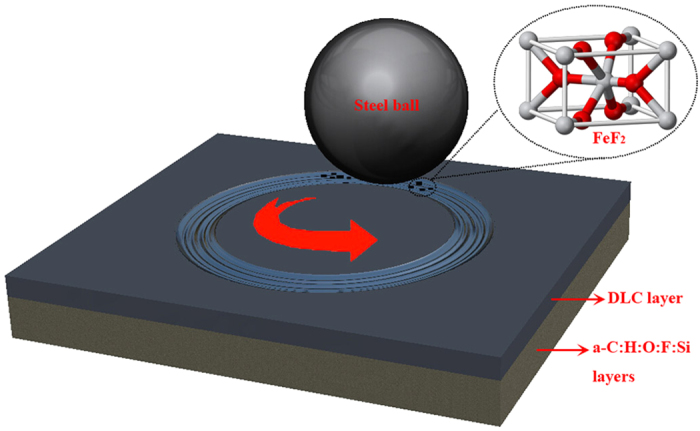
Schematic diagram showing the frictional mechanism responsible for the amorphous carbon film against steel ball under high vacuum.

Although, hydrogenated, fluorinated and Ag or Al doped amorphous carbon films exhibited superior tribological properties under vacuum^10^,^11^,^33^,^34^, ultra-long wear life of these films had been rarely reported, to the best our knowledge. Thus, in this investigation, based on this aim, F and Si codoped amorphous carbon film is successfully fabricated on stainless steel substrates using a plane hollow cathode plasma-enhanced chemical vapor deposition method. From the XPS spectra (Figure 3) obtained from original film, F exists in the form of C−F bonds. Two principal results pertaining to the tribological behavior of amorphous carbon film have been obtained in this paper. The first is that the steady-state friction coefficient and ultra-low wear is observed, and the second is evidence for F transfer to the steel ball counterface. And F accumulates in the wear track could also be related to the more stable network of C-F bonds compared to amorphous carbon. The detection of F-containing transfer layer using XPS, EDS and EELS and the evidence for the formation of thermodynamically and structurally stable FeF_2_ at the transfer layer generates at the contact surfaces supported the key predictions of the first-principles calculations. Results of first-principles calculations and sliding experiments, combining with the high value of adhesion load and hardness, although not performed under identical contact and environmental conditions, complement each other, and when analyzes together they could depict a coherent picture of intrinsic tribological mechanism. Accordingly, a model that accounts for the tribological mechanism between amorphous carbon film and steel ball surfaces is proposed as shown in Figure 15.

According to the analysis in Figures 4, and 6, it shows the recombination of bonds and atoms towards the formation of FeF_2_ nanocrystallite. The most plausible mechanism would be a re-arrangement of the bonds from C−F to Fe−F during sliding. Although graphitization occurs at the wear track (Figure 11), combining with Figure 9, the outer FeF_2_ nanocrystallite-containing crystal layer just separate the amorphous carbon transfer layer and the film, avoiding the occurrence of strong adhesion between two contact surfaces under high vacuum. As we known, the strong adhesion between two contact surfaces would result in high friction and wear under high vacuum, which led to the fast failure of the film, as reported in the previous study^35^. Thus, the formation of FeF_2_ nanocrystallite results in ultra-low wear and ultra-long wear life in high vacuum.

### Experimental procedure

#### Film preparation

The film was deposited on AISI 304 stainless steel substrates (30 mm × 30 mm × 2 mm) by a plane hollow cathode plasma-enhanced chemical vapor deposition method. The AISI 304 stainless steel substrates were polished to a mirror finish. The deposition process was proposed by our previous studies^12^,^13^. Before deposition, stainless steel substrates were ultrasonically cleaned in acetone and absolute ethanol for 40 min, respectively. Then the base pressure of depositing chamber was pumped down to 5.0 × 10^−3^ Pa. Substrates were cleaned at a pressure of 1.5 Pa for 15 min with a constant flow of argon (Ar) gas. Before film deposition, a Si interlayer of about 0.2 μm was deposited with SiH_4_ gas of 50 sccm (–15.0 kV bias voltage, 15 Pa and 30% duty ratio) to improve adhesion of the final film to substrates. The film consisting of (F_*x*1_-Si_*y*1_–DLC/F_*x*2_-Si_*y*2_–DLC)_n_ was fabricated by repeated synthesis of F_x1_-Si_y1_–DLC (low F and Si doped DLC layer) and F_x2_-Si_y2_–DLC (high F and Si doped DLC layer, *x*1,*y*1 < *x*2,*y*2); n was the number of (F_x1_-Si_y1_–DLC/F_x2_-Si_y2_–DLC) layers, in this paper, the value of n was set to 16. The F_x1_-Si_y1_−DLC layers were deposited in SiH_4_ (25 sccm), CF_4_ (25 sccm), C_2_H_2_ (150 sccm) and Ar (100 sccm) at 4.0 Pa. The F_x2_-Si_y2_−DLC layers were deposited in SiH_4_ (25 sccm), CF_4_ (25 sccm), C_2_H_2_ (100 sccm) and Ar (100 sccm) at 2.8 Pa. The a-C:H film was deposited on a Si interlayer about 200 nm in C_2_H_2_ and Ar environment at 3.4 Pa with a total gas flow of 150 sccm and 100 sccm. The substrate bias voltage was maintained at −800 V, a duty cycle of 30%, and a repetition frequency at 1.5 kHz. No external heating of the substrate was employed, and the maximum temperature during deposition was about 180 °C.

#### Film characterization

TEM specimens were extracted from the studied coating as follows. A suitable region for side-view imaging is selected by using a thermal field electron emission scanning electron microscope (FEISEM;). A Pt strap was deposited over the whole surface. The metal layer was employed to prohibit charging and protect the selected region from ion damage^36^. Focused ion beam (FIB) milling was used to fabricate a cross-sectional slice under the Pt strap. The slice was then transferred and attached to a TEM grid, this sample preparation method known as the lift-out methods^37^,^38^. Finally, the low-energy ion milling was selected to thin the extracted slice down to about 60 nm, it ould be then analyzed at high-resolution transmission electron microscopy (HRTEM). The microstructure of transfer film formed on the worn surface of GCr 15 steel ball and cross-sectional of the wear track was examined in details by high-resolution transmission electron microscopy (HRTEM; TF20). The electron energy loss spectra (EELS) were obtained using a JEOL 2010F operated at 197 kV and equipped with a Gatan GIF 200 spectrometer. The fracture cross-sectional microstructure of the coating was obtained by a thermal field electron emission scanning electron microscope (FEI Quanta FEG 250). An Ar^+^ ion source provided for sputter-etch cleaning of specimens. The chemical composition of transfer film formed on the surface of GCr15 steel ball and the uppermost part of the amorphous carbon film was examined using X-ray photoelectron spectroscopy (XPS: AXIS ULTRA ^DLD^). All the fitting XPS C1s, F1s, O1s and Fe2p spectra were performed by approximating the contribution of the background by the Gaussian method. The measurement was carried out using 36 MeV iodine ions as incoming ion projectile. The spectrometer was calibrated using a standard silver sample. Data were processed by Specslab2 software. The GIXRD patterns of the films were obtained using GIXRD modes with the X-ray diffractometer (Bruker D8 Discover, Germany). Cu Kα (λ = 1.5406 Å) was used as the source. The diameter of beam spot was 15 μm. Auger electron spectroscopy (AES) was used to characterize the amorphous carbon film. Hydrogen content was determined using time-of-flight elastic recoil detection analysis (TOF-ERDA). Raman spectra of the amorphous carbon film were obtained by a Horiba Jobin Yvon LABRAM-HR800 spectrometer using an excitation wavelength of 532 nm. The typical spectrum was recorded in the range of 800–2000 cm^−1^, data acquisition time was 60 s and diameter of beam spot was 0.2 μm. The non-contact 3D surface profiler images of the amorphous carbon film sliding against stainless steel ball were performed using AEP non-contact 3D surface profiler. The curvature radii of the substrate before and after coating deposition were measured by the observation of Newton’s rings using an optical interferometer system, and then the residual stress was calculated by the Stoney equation. The adhesion of the sample was tested by a scratch tester (CSEM Revetest) equipped with a diamond tip of radius 200 μm. The normal load was increased from 0 to 100 N at a loading rate of 100 N/min and a scratching speed of 10 mm/min. The hardness and elastic modulus was measured by a Nanotest 600 nanoindenter apparatus using a Berkovich diamond tip and continuous stiffness option, and the maximum indentation depth was limited to 1/10 of the film thickness to minimize the substrate contribution. The hardness and elastic modulus were obtained using the Oliver–Pharr method. The frictional behavior of the sample was performed on a vacuum tribo-meter with a frictional force sensor with an accuracy of 5 mN, using a rotational ball-on-disc mode. The friction force sensor is implemented on the balance bar. Tribological tests were done under vacuum ranged from 5.0 × 10^–2^ to 1.0 × 10^–4^ Pa. The rotational speed was 300 rev min^–1^, and the rotational radius was set to 4.0 mm. The corresponding linear speed was about 0.125 m s^–1^. The counter face was standard GCr15 steel ball with 4 mm diameter. The normal load was set as 2.0 N, corresponding to a theoretical initial Hertzian contact pressure of 0.62 GPa. The load (normal force) is added on the tribometer through equal amounts of weight. The two surfaces are in contact during pumping.

### Computational procedure

The CASTEP module in the Materials Studio 5.5 program of Accelrys Inc was used to calculate the ground state energy and geometry of each interface. The exchange-correlation energies were calculated using the Perdew, Burke, and Ernzerhof (PBE) functional^39^ with the generalized gradient approximation (GGA)^40^. A plane-wave cutoff of 360 eV and Monkhorst-Pack *k*-point meshes with a density of (7 × 7 × 1) was employed throughout. For comparison, the total energies of Fe and fluorine terminated diamond (FTD) interface was calculated with GGA. The electron-ion interactions were described by ultrasoft pseudopotentials. To confirm the accuracy of our calculations, the total energies of Fe and FTD interfaces were first calculated by taking the convergence tests and the spin-polarization into consideration. A Fermi smearing of 0.1 eV was utilized. The convergence criteria for structure optimization and energy calculation were set to MEDIUM quality with the tolerance for SCF, energy, maximum force, and maximum displacement of 2.0 × 10^–6^ eV/atom, 2.0 × 10^–6^ eV/atom, 0.05 eV/Å, and 2.0 × 10^–3^ Å. In this article, the tribological surface of DLC coating was represented by a diamond surface such as (111), as declared by Guo^41^. The Fe(111)–1 × 1/FTD(111)–1 × 1 interface was used in all calculations. Fcc Fe was selected to minimize the lattice mismatch of Fe(111)–1 × 1/FTD(111)–1 × 1 interfaces, this corresponded to an average lattice mismatch of 1%. After geometrical optimizations, the lattice parameters of fcc Fe were as follows: 3.40 Å. They were in well consistency with experimental values: 3.45 Å^42^. It indicated that the calculation methods were reasonable and the calculation results should be authentic. A slab model was used to simulate the geometries of the Fe and the FTD surfaces. The slabs consisted of 5–10 layers of Fe atoms separate by a 10 Å vacuum gap. The minimum number of atomic layers was necessary for simulating the convergence in surface energies for slabs replacing Fe and FTD surfaces individually. The surface energy of Fe (111) surface contained one Fe atom per layer and converged at –8657.48 eV in ten layers of empty spheres. It allowed us to vary the separation between phases without changing the cell size. Calculations carried out to estimate the surface stability of FTD surface, indicating that a slab with six layers would be adequate^43^.

The interface model was shown in Figure S2, and consisted of a periodic arrangement of alternating FTD and Fe layers with 15 vacuum layers that have, at minimum, the inversion symmetry to ensure that both interfaces were the same. Interface calculations were carried out under vacuum at 0 K and the effects of plastic deformation and diffusion did not take into consideration. Initially, atoms on Fe and FTD surface were placed 8.6 Å apart, and then the interfacial separation distance (*d*_Fe–F_) was gradually decreased to 0.8 Å. The total energy of system (*E*_tot_) was calculated by letting the atoms relax in their initial positions without allowing the constrained interface structure and atoms to relax. The change of *E*_tot_ corresponded to be relative to that of reference state *E*_tot′_ at the far separated interface of *d*_Fe–F_ = 8.6 Å. The relative energy change 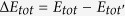 was for the Fe and FTD interface. Then the work of separation (*W*_sep_) could be computed via
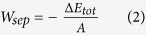
where *A* represented the interface area.

## Additional Information

**How to cite this article**: Wang, L. *et al.* A near-wearless and extremely long lifetime amorphous carbon film under high vacuum. *Sci. Rep.* **5**, 11119; doi: 10.1038/srep11119 (2015).

## Supplementary Material

Supplementary Information
