# Supplementary material for: A near-wearless and extremely long lifetime amorphous carbon film under high vacuum
Source: Sci Rep. 2015 Jun 10;5:11119. doi: 10.1038/srep11119 (PMC4461914; doi:10.1038/srep11119)
Supplement: Supplementary Information [file srep11119-s1.doc]

**Supporting Information**

**A near-wearless and extremely long lifetime amorphous carbon coating under high vacuum**

Liping Wang*^1^, Renhui Zhang^1,2^, Ulf Jansson^3^, Nils Nedfors^3^

*^1^State Key Laboratory of Solid Lubrication, Lanzhou Institute of Chemical Physics, Chinese Academy of Science, Lanzhou 730000, China, ^2^University of Chinese Academy of Sciences, Beijing 100039, China, ^3^Department of Materials Chemistry, The Ångström Laboratory, Uppsala University, box 538, SE-751 21 Uppsala, Sweden.*

*Correspondence: Professor L Wang, State Key Laboratory of Solid Lubrication, Lanzhou Institute of Chemical Physics, Chinese Academy of Sciences, 18 Tianshui Middle Road, Lanzhou, Gansu 730000, China.

E-mail: [lpwang@licp.cas.cn](mailto:lpwang@licp.cas.cn))

Supporting Information includes:

- Figure S1 ………………………………………………………… Page S2
- Figure S2 ………………………………………………………… Page S2
- Figure S3 ........................................................................................ Page S3
- Figure S4 ........................................................................................ Page S4
- Figure S5 ………………………………………………………… Page S4
- Figure S6 ..……………………………………………………….. Page S5
- Table S1............................................................................................Page S6
- Figure S7 .........................................................................................Page S6
- Figure S8 .........................................................................................Page S7
- References ……………………………………………………….. Page S9

**
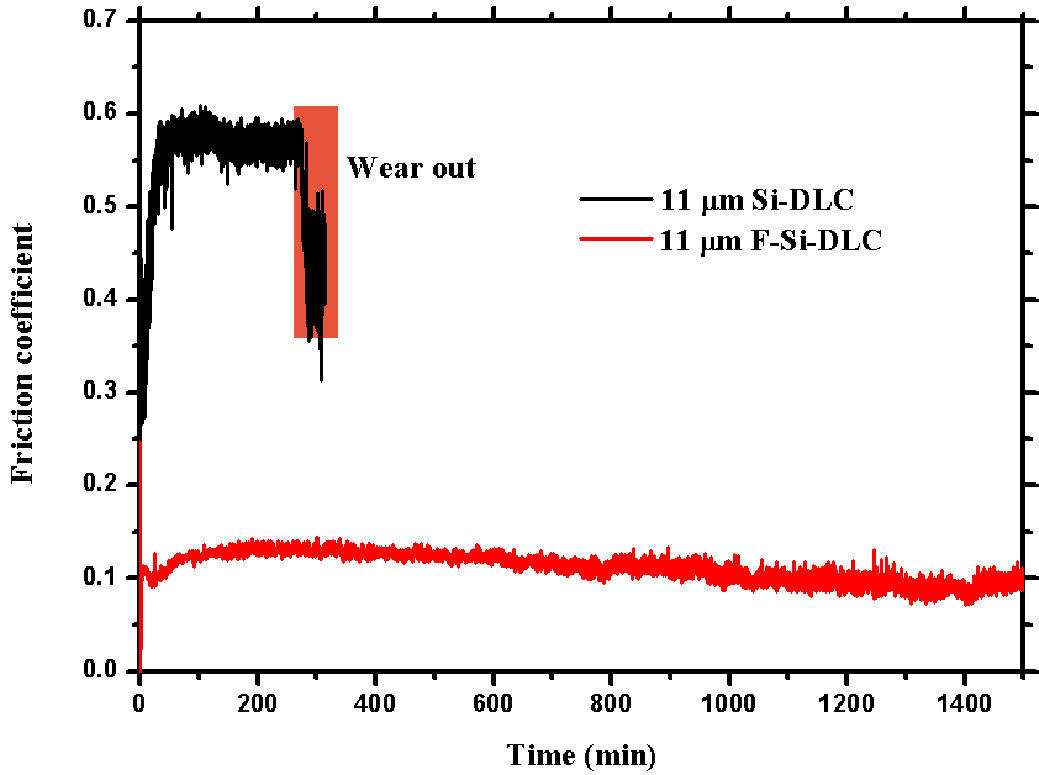
**

**Figure S1** Friction coefficient of F-Si-DLC and Si-DLC films under vacuum (1.0 × 10^-3^ Pa).


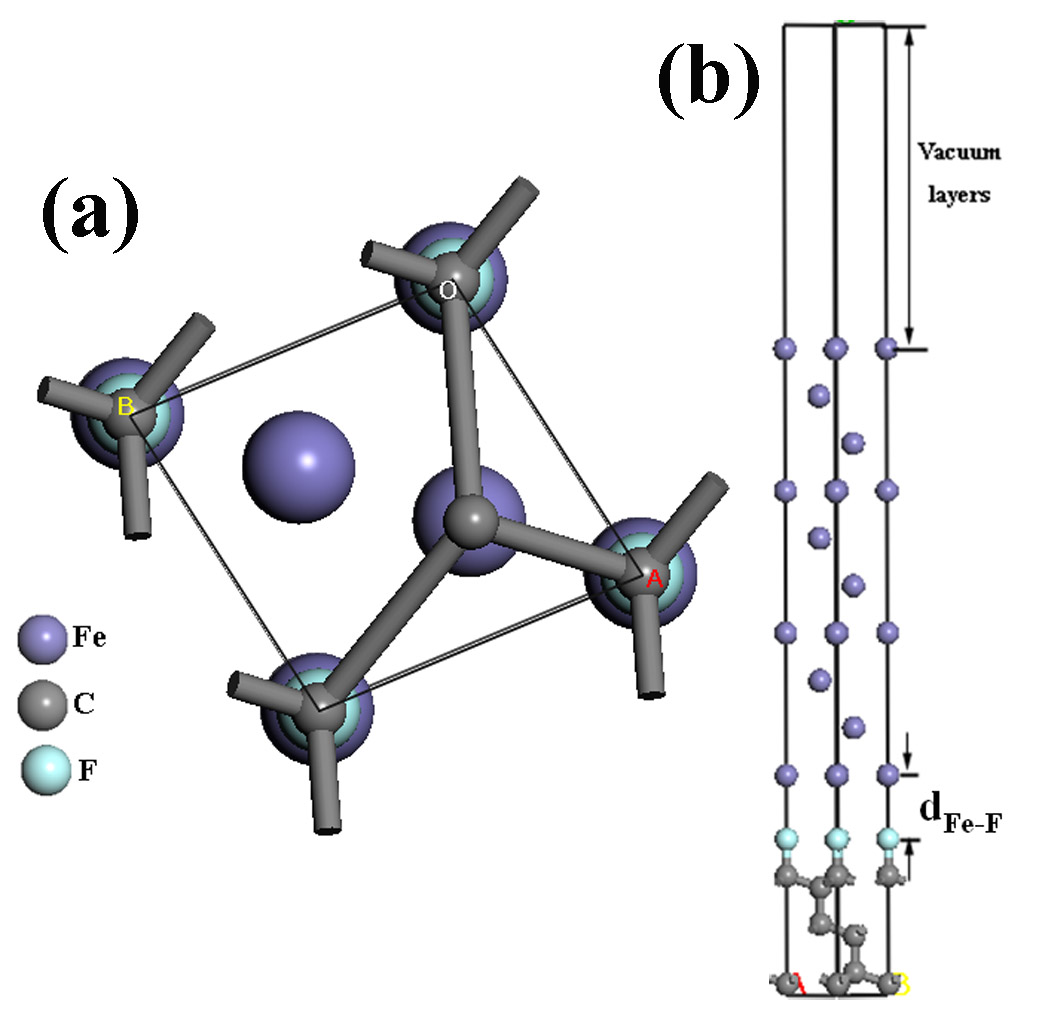


**Figure S2** Fe and FTD interface model employed in DFT calculations. (a) Top view of the interface registry, where the edge length of the cell is 2.50 Å. (b) Side view of the interface model formed between 10 layers of Fe and 6 bilayers of FTD, ten vacuum layers are added in this model. *d*_Fe–F_ is the distance between the Fe and F atom at the interface.

**
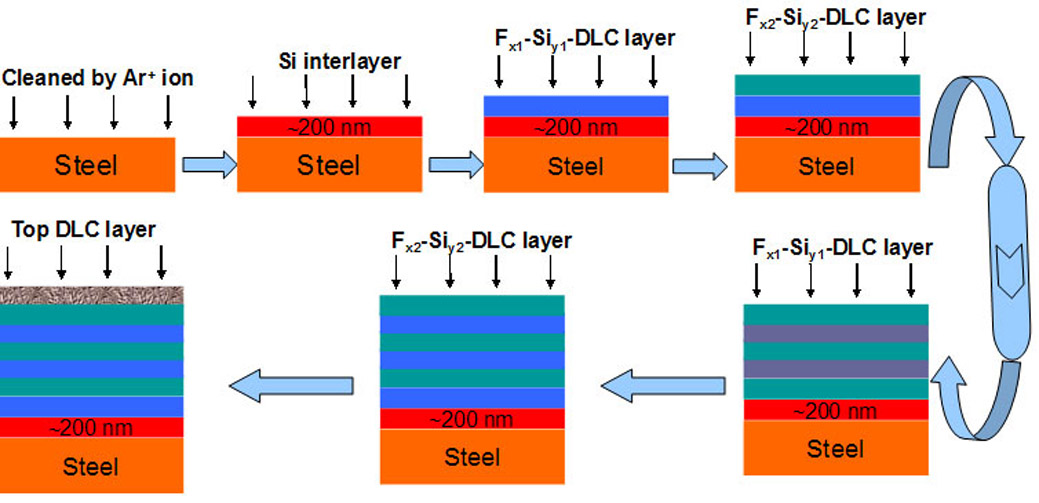
**

**Figure S3** The deposition processes of the film.


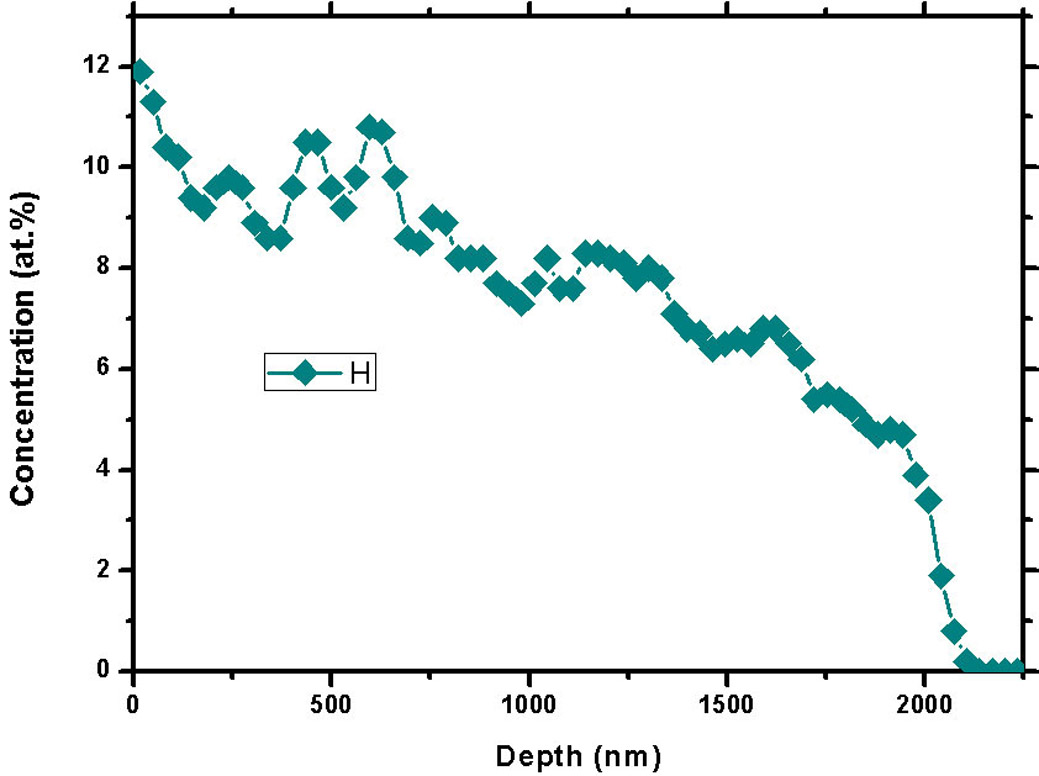


**Figure S4** The hydrogen concentration of the amorphous carbon film examined using time-of-flight elastic recoil detection analysis.


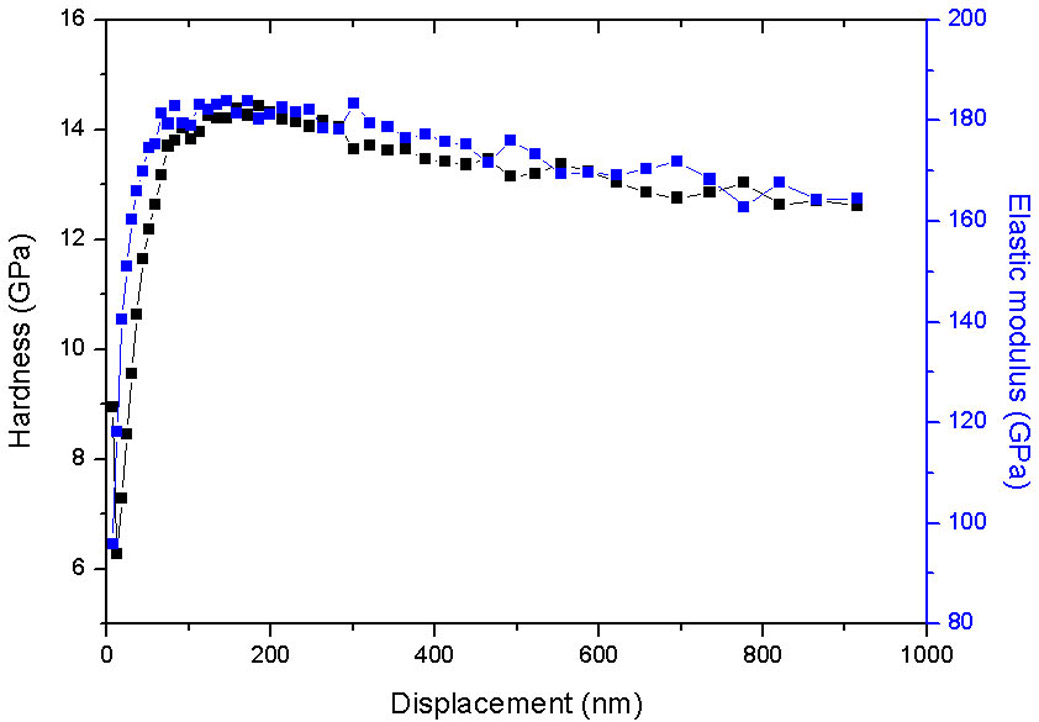


**Figure S5** The evolution of the hardness and elastic modulus as a function of depth of indent for the amorphous carbon film.


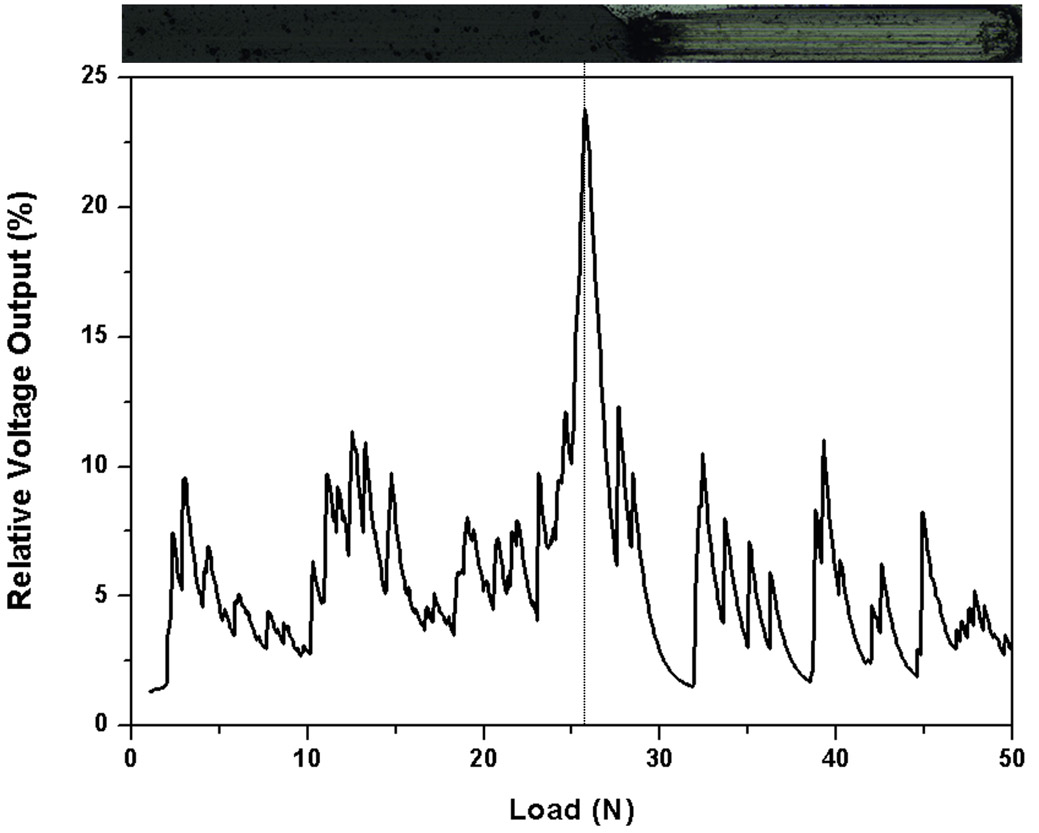


**Figure S6** The scratch curve of the amorphous carbon film.


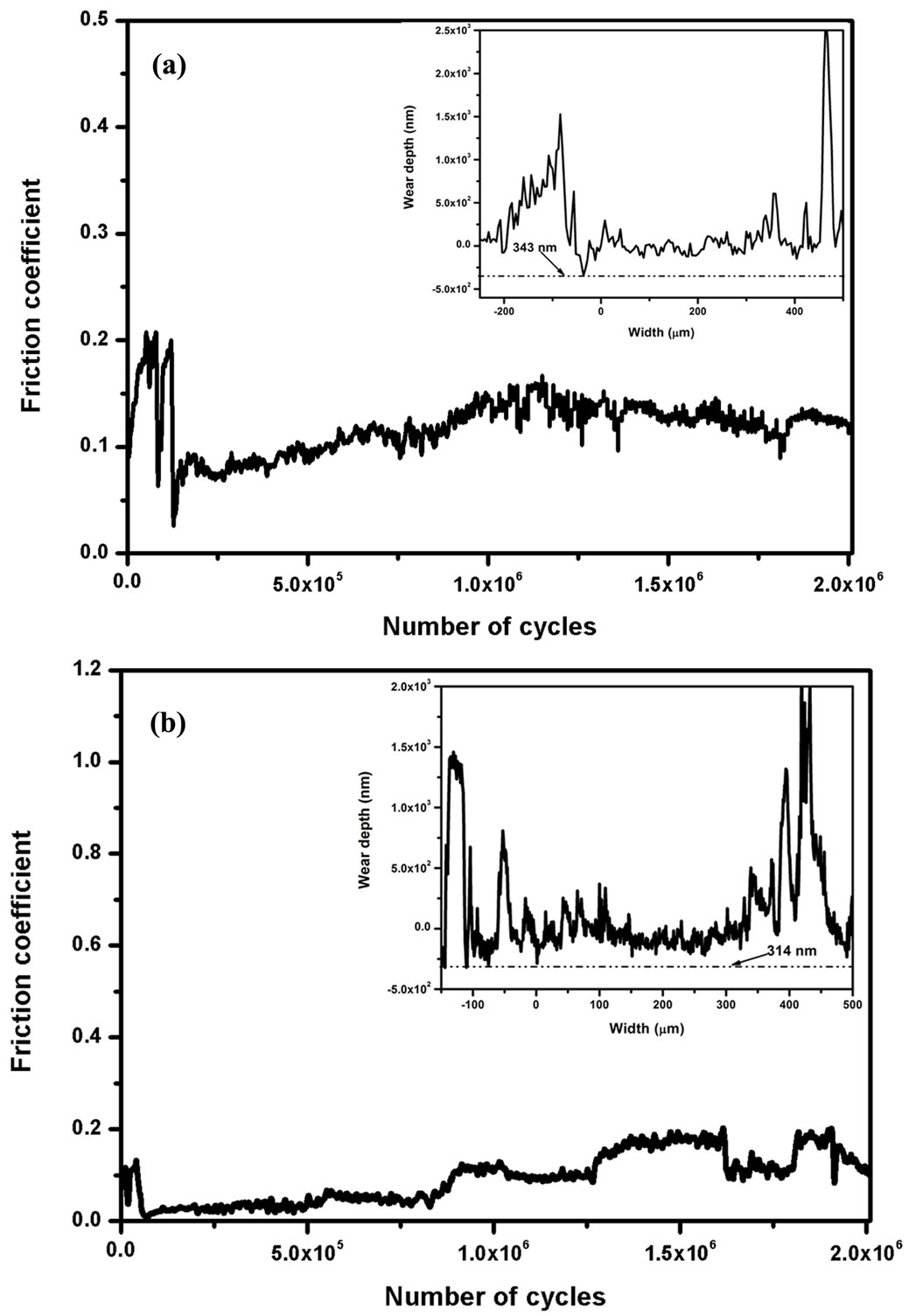


**Figure S7** Repeat tribological experiments of amorphous carbon film against GCr15 steel ball for two million sliding cycles.

**Table S1.** The wear rate, maximum wear depth, number of cycles of amorphous carbon film against GCr15 steel ball for more than two million sliding cycles.

| Number | Wear depth (nm) | Wear rate (mm^3^ N^-1^ mm^-1^) | Number of cycles |
| --- | --- | --- | --- |
| 1 | ~ 360 | 9.0 × 10^-13^ | > 2 × 10^6^ |
| 2 | ~ 345 | 7.6 × 10^-13^ | > 2 × 10^6^ |
| 3 | ~ 320 | 8.2 × 10^-13^ | > 2 × 10^6^ |


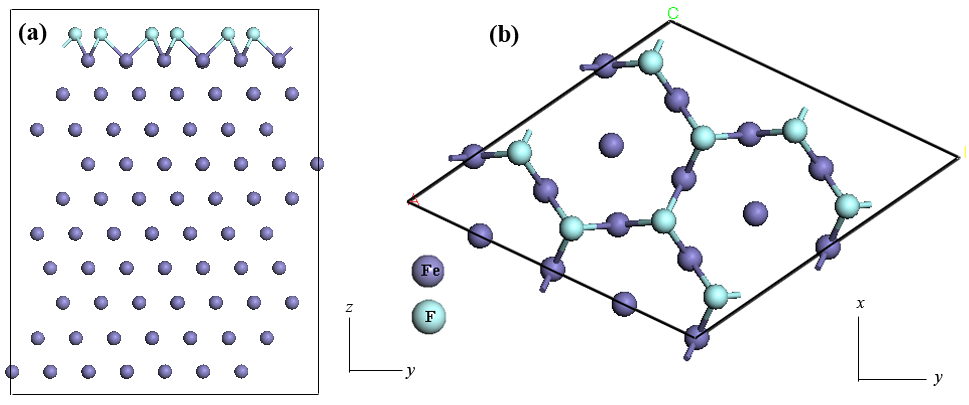


**Figure S8** Reconstructed Fe (111) surface after transferring two F atoms. (a) Side view that shows the Fe surface undergoes severe deformation. The Fe−F bond distance is 1.94 and 2.01 Å. The F–Fe–F is 106.3° and 126.8°, and angle Fe–F–Fe is 90°. (b) Top view of reconstructed Fe (111) surface after the transfer of 2 F atoms.

To further confirm the stability of the formed FeF_2_ compound, the Fe (111)-2×2 and FTD (111)-2×2 interface is selected to illustrate the formation of FeF_2_. The formation of this compound is an important aspect of the material transfer mechanisms at the Fe and FTD interfaces. The atomic configuration of 2 F-transferred Fe surface is illustrated in **Figure S8**. **Figure S8**a shows the side view of the Fe surface after two F transfer. The Fe (111) surface undergoes a severe plastic deformation. The dislocations are found between Fe layers during the reconstruction of the Fe surface. The atomic configuration of 2 F-transferred Fe surface is presented in **Figure S8**b, suggesting the formation of FeF_2_ compound. The bond structure of 2 F-transferred Fe surface is compared with the bond structure of FeF_2_ compound by relaxing FeF_2_ crystal. Then we calculated the lattice parameters of the thermodynamically stable structure, which belongs to space group P42/mnm. In this primitive cell, the structure of FeF_2_ can be viewed as edge-sharing FeF_6/3_ octahedra.^1^ The lattice parameters of the relaxed FeF_2_ unit cell is calculated as *a* = 4.459 Å, *c* = 3.226 Å and *c*/*a* = 0.723, which are in consistence with the reported experimental values.^2^ In this structure, the Fe–F bond distance is 1.94 Å and 2.01 Å and angle F–Fe–F is 106.3° and 126.8°, and angle Fe–F–Fe is 90°. As comparing the FeF_2_ crystal with the 2 F-transferred Fe surface, The Fe–F bond distance (2.04 ± 0.01Å) and F–Fe–F angle (87.4°) values prove to be in good agreement. Differently, the calculated Fe–F–Fe angle is higher than that of the FeF_2_ compound, because no octahedral are presented in the reconstructed Fe surface. And a thermodynamic argument can be done to support the formation of FeF_2_. This compound is formed as a result of the following reaction:

$4F\text{e}+C_{48}F_{8}=C_{48}+4F\text{e}F_{2}$ (1)

The enthalpy of formation for FeF_2_,$H_{FeF_{2}}^{f}$, can be calculated as follows:

$H_{FeF_{2}}^{f}=\frac{1}{4}[E_{C_{48}}+4E_{FeF_{2}}-E_{C_{48}F_{8}}-4E_{Fe}]$ (2)

where $E_{C_{48}}$and $E_{C_{48}F_{8}}$represent the total energies of the six bilayers of diamond (111)-2 × 2 slabs with and without F termination. $E_{Fe}$is the energy of one atom in a bulk Fe face-centered cubic unit cell. $E_{FeF_{2}}$represents the energy of one FeF_2_ molecule, calculated in the FeF_2_ crystal. Finally, $H_{FeF_{2}}^{f}$can be calculated as the value of –31.3 kJ mol^-1^. The negative formation energy indicates that the formation of FeF_2_ is thermodynamically and structurally stable.^3^

**References**

1. Ma, Y., Lockwood, G.K. & Garofalini, S.H. Development of a transferable variable charge potential for the study of energy conversion materials FeF_2_ and FeF_3_. *J. Phys. Chem. C* **115**, 24198–24205 (2011).

2. Kestigian, M., Leipziger, F.D., Croft, W.J. & Guidoboni, R. Single crystal growth, and crystallographic properties of FeF_2_, RbFeF_3_, and CsFeF_3_. *Inorg. Chem.* **5**, 1462–1463 (1966).

3. Odkhuu, D., Shin, D., Ruoff, R.S. & Park N. Conversion of multilayer graphene into continuous ultrathin sp3-bonded carbon films on metal surfaces. *Sci. Rep.* **3**, 1–7 (2013).
